# Validity of a questionnaire measuring the world health organization concept of health system responsiveness with respect to perinatal services in the dutch obstetric care system

**DOI:** 10.1186/s12913-014-0622-1

**Published:** 2014-12-03

**Authors:** Jacoba van der Kooy, Nicole B Valentine, Erwin Birnie, Marijana Vujkovic, Johanna P de Graaf, Semiha Denktaş, Eric AP Steegers, Gouke J Bonsel

**Affiliations:** Department of Obstetrics and Gynaecology, Division of Obstetrics & Prenatal Medicine, Room Hs-408, Erasmus MC, PO Box 2040, Rotterdam, CA 3000 The Netherlands; World Health Organization, Social Determinants of Health - Public Health, Environmental and Social Determinants of Health, Family, Women’s and Children’s Health, Avenue Appia 20, Geneva, 1211 Switzerland; Institute of Health Policy and Management, Erasmus University Rotteram, PO Box 1738, Rotterdam, DR 3000 The Netherlands; University of Applied Sciences, Midwifery Academy Rotterdam (Verloskunde Academie Rotterdam), Dr. Molewaterplein 40, Rotterdam, GD 3015 The Netherlands; Department of Public Health, Erasmus MC, PO Box 2040, Rotterdam, CA 3000 The Netherlands

**Keywords:** Responsiveness, Psychometric properties, Quality of care, Perinatal care

## Abstract

**Background:**

The concept of responsiveness, introduced by the World Health Organization (WHO), addresses non-clinical aspects of health service quality that are relevant regardless of provider, country, health system or health condition. Responsiveness refers to “aspects related to the way individuals are treated and the environment in which they are treated” during health system interactions. This paper assesses the psychometric properties of a newly developed responsiveness questionnaire dedicated to evaluating maternal experiences of perinatal care services, called the Responsiveness in Perinatal and Obstetric Health Care Questionnaire (ReproQ), using the eight-domain WHO concept.

**Methods:**

The ReproQ was developed between October 2009 and February 2010 by adapting the WHO Responsiveness Questionnaire items to the perinatal care context. The psychometric properties of feasibility, construct validity, and discriminative validity were empirically assessed in a sample of Dutch women two weeks post partum.

**Results:**

A total of 171 women consented to participation. Feasibility: the interviews lasted between 20 and 40 minutes and the overall missing rate was 8%. Construct validity: mean Cronbach’s alphas for the antenatal, birth and postpartum phase were: 0.73 (range 0.57-0.82), 0.84 (range 0.66-0.92), and 0.87 (range 0.62-0.95) respectively. The item-own scale correlations within all phases were considerably higher than most of the item-other scale correlations. Within the antenatal care, birth care and post partum phases, the eight factors explained 69%, 69%, and 76% of variance respectively. Discriminative validity: overall responsiveness mean sum scores were higher for women whose children were not admitted. This confirmed the hypothesis that dissatisfaction with health outcomes is transferred to their judgement on responsiveness of the perinatal services.

**Conclusions:**

The ReproQ interview-based questionnaire demonstrated satisfactory psychometric properties to describe the quality of perinatal care in the Netherlands, with the potential to discriminate between different levels of quality of care. In view of the relatively small sample, further testing and research is recommended.

**Electronic supplementary material:**

The online version of this article (doi:10.1186/s12913-014-0622-1) contains supplementary material, which is available to authorized users.

## Background

The debate on the organization of perinatal care in the Netherlands has intensified over recent years. The Dutch perinatal health care system has come under pressure since the national perinatal mortality rates were shown to be among the highest in Europe [1]. This system can be regarded as a sequential chain of health care services, each dedicated to a different phase of the perinatal experience: antenatal care, birth and post partum care. Antenatal, birth-related and post partum care are provided by different caregivers with different responsibilities, for different risk groups, and in different settings. In the Netherlands independently operating community midwives provide care for low-risk pregnant women (primary healthcare) while gynecologists provide in-hospital care for high-risk women (secondary care). All women receive post partum care by a community midwife.

The performance of perinatal care is often judged by its endpoints such as clinical outcomes and costs. However, quality of care literature supports the view that non-clinical aspects of quality are important too, and affect clinical outcomes [2-4]. Better service quality is thought to increase compliance with medical treatment and to improve the transfer of information and appropriate utilization of health care [5-8]. Governments of Western countries increasingly acknowledge the importance of the non-clinical aspects of quality of care and incorporate these when the provision of care is monitored [9,10].

Sofar, no attempts have been made to evaluate the non-clinical aspects of the Dutch perinatal care system such that not only the heterogeneity in the quality with respect to different perinatal services is identified, but also that international comparisons with other obstetric care systems are possible [11-13]. The concept of responsiveness, introduced by the World Health Organization (WHO) in 2000, seems apt to this task as it was specifically developed to refer to patients’ experiences when interacting with health care providers. The concept reviews eight predefined domains relevant to non-clinical aspects of service quality regardless of provider, country, health system or health condition. Responsiveness is defined as “aspects related to the way individuals are treated and the environment in which they are treated during health system interactions”, encompassing the notions of both non-clinical quality and patient experience [14]. The concept excludes the financial and clinical domains of quality and focuses on a set of non-clinical domains that reflect respect for human dignity and the client orientation of the care process and setting. While these domains may influence health outcomes, health outcomes are not part of the responsiveness concept. The relevance of an independent set of non-clinical domains to health systems performance is supported by the discipline of medical ethics and in human rights law, which argue that responsiveness features of a health system are important in their own right [14-16]. The concept of responsiveness aims to support measurement of service quality in an internationally comparable way and to enable quantitative trade-offs between non-clinical aspects of service quality and clinical outcomes [14]. The concept of responsiveness aims to capture information on the non-clinical quality of the patient’s actual experience in contrast to patient satisfaction questionnaires. Literature has shown that expectations may strongly influence patient satisfaction, which makes international comparisons of non-clinical service quality challenging since expectations are in turn influenced by economic and political influences [17-20].

Adopting the responsiveness concept, the Responsiveness in Perinatal and Obstetric Health Care Questionnaire questionnaire (ReproQ) was developed by adapting the existing generic World Health Survey questionnaire responsiveness module into a questionnaire dedicated to maternal experiences during perinatal care. The aim of this study is to investigate the psychometric properties of the ReproQ.

## Methods

### Questionnaire

The WHO developed a survey, which was administrated between 2000–2001 under the auspices of the Multi-Country Survey Study on Health and Health Systems Responsiveness (MCS Study) and again in 2002–2003 under the World Health Survey (WHS) [14,21]. The concept of responsiveness, containing eight domains, was identified in WHO’s review of the patient satisfaction and quality of care literature [15].

Several questionnaires and related studies relevant to responsiveness domains were used, such as the Community Tracking Study [22], Picker Survey [23], QUOTE study [24] and the CAHPS (Consumer Assessment of Health Plans Study) [25].

Although there are many overlapping aspects with patient satisfaction questionnaires, the concept of responsiveness is different on several points; where patient satisfaction generally covers both medical and non-medical aspects of care responsiveness focuses only on the non-clinical aspects of the health system. Where patient satisfaction represents a complex mixture of perceived need, individually determined expectations and experience of care, responsiveness evaluates individual’s perceptions of the health system against ‘legitimate’ expectations – referring to standards that can be applied everywhere or ‘universally’ [15].

The ReproQ was developed between October 2009 and February 2010, and its questions were derived from these WHO questionnaires.

The ReproQ questionnaire was developed to assess the responsiveness outcomes of perinatal health care system in the Netherlands and is based on the same eight domains identified in WHO’s review, i.e. Dignity, Autonomy, Confidentiality, Communication, Prompt Attention, Social Consideration (labeled initially as *Access to Social Support* or *Access to Family and Community Support*), Quality of Basic Amenities, and Choice and Continuity.

These domains are claimed to be of universal importance in all health systems, during any client-system interaction (including personal and non-personal health services) and for the population’s interaction with insurers and other administrative arms of the health system. While it is recognized that persons may differ regarding the relative importance of each domain, and that specific domains may be of extra relevance in particular health care interactions, it is assumed that the quality of any health care interaction is sufficiently covered by these eight domains [14].

The ReproQ asks the same questions for the three phases of perinatal care: antenatal phase (the period from the onset of pregnancy until the onset of delivery), birth phase (actual delivery) and post partum phase (covering the first ten days after childbirth). Rather than pointing to a single event, or the last visit (as in the WHS), we selected to focus questions on women’s judgments for all antenatal visits as done for the MCS Study. The ‘last’ visit approach has better recall but is easily biased by a particular incident. We wanted to review the experience as a whole and thought the multiple visit approach more suited to this. A similar argument applied to the decision to focus postnatal maternity care questions on all visits. For the birth phase, it seemed appropriate to focus questions on the single event of ‘delivery’. Within this framework, the setting and professional items where adapted to the perinatal care in the obstetric care system (e.g. ‘doctor’ was translated into ‘midwife’ or ‘gynecologist’). If two different health care professionals could be involved (e.g. ‘midwife’ and ‘nurse’ during delivery), similar questions within each domain were repeated for each health care professional separately.

Each phase was covered by the above mentioned eight domains, with 2–7 items per domain. The standardized response mode consisted of 5 options: ‘very good’, ‘good’, ‘moderate’, ‘bad’, and ‘very bad’. The ReproQ consisted of 104 questions on responsiveness (25 antenatal, 40 birth, 39 postpartum phase) and 29 questions for maternal and health care characteristics.

Questions from the WHO questionnaire were translated into Dutch according to a predefined protocol. First, questionnaires were translated by the research team. Expert meetings consisting of gynecologists, midwives, nurses, public health experts and researchers were held to judge the translation and comprehensiveness of the item list. Many among these professionals had working experience in English speaking countries. Next, backward translation of each question was then performed and comparison was made with the original English questionnaire. Improvements were made and final consensus was reached on each question.

The completeness of domains was judged in terms of being comprehensive (are all non-clinical areas covered, which clients and professionals put forward either as positive experience or negatively as complaint), and in terms of being balanced (have all domains included about equal importance). For each domain the candidate pool of items was checked whether each item fitted to the domain definition sufficiently. As this could differ per phase, this was discussed for each phase separately (e.g. the item ‘quality of the food’ during antenatal visits was excluded). Finally we asked the experts to check whether all the domains would remain valid under ongoing and anticipated organizational changes in perinatal care. All experts agreed on the final list that the stated requirements were met.

Finally six primiparous and multiparous pregnant women were invited to judge the feasibility of the draft version of the questionnaire. Since we adopted an existing concept and adapted existing questions from an extensively studied source questionnaire towards a perinatal context, we invited the judgement of these six women in the final stage. They were first asked to conduct a brainstorm on important non- clinical aspects of perinatal care. Next, the ReproQ was evaluated to see whether its domains covered these issues. All items were discussed separately including their meaning and understandability. The original domain structure proved to be comprehensive, as judged by the stakeholders. Small textual improvements were made in the item questions as a result of this meeting Table 1.

**Table 1:** **Shows the eight domains and items for the antenatal phase**

Dignity	Were physical examinations and treatments done in a way that respected your privacy?
	Did the examination rooms ensure your privacy?
	Were you treated with respect by your health care provider?
Autonomy	How well were you involved in making decisions regarding your examinations or treatments?
	Were you able to refuse examinations or treatments?
	Were you asked permission before testing or starting treatment?
Confidentiality of Information	Were consultations carried out in a manner that protected your confidentiality?
	Was confidentiality kept on the information provided by you?
	Was your medical record kept confidential?
Communication	How well were things explained by your health care provider in a way you could understand?
	Was written information provided in such a way you could understand?
	Were you encouraged to ask questions about your health problems, treatment and care?
	Were you given time to ask questions about your health problem or treatment?
	Was information on the health service’s contact, location and parking information clear to you?
Prompt Attention	How well did you receive prompt attention at your health service?
	How did you experience the waiting time after you asked for help?
	How well was the accessibility by phone?
	How do you rate the travel time to your health service?
Social Consideration	Did the health care provider facilitate the support of your relatives and friends?
	Was the home situation taken into consideration when planning an appointment?
Quality of basic amenities	How do you rate the quality of the hygiene of the toilets?
	How do you rate the overall quality of the surroundings, for example, space, seating, fresh air and cleanness?
Choice and continuity of health care provider	Were you able to choose your own health care provider?
	Were you able to use other health care services other than the one you usually went to?
	How well was the continuity of care by one health care provider?
	Were you able to choose your own place of delivery?

### Study population and data collection

Study approval was granted by the Medical Ethical Committee, Erasmus Medical Centre, Rotterdam, the Netherlands, no MEC2012207.

To investigate the psychometric properties of responsiveness questions for each phase, women were recruited from three midwifery practices in Rotterdam, the Netherlands, between February 2010 and March 2011 (all women, regardless their health utility, receive post partum care by a community midwife in the Netherlands). Women or their partners were required to speak and understand Dutch sufficiently. Written informed consent was obtained.

The survey was administered in the form of face-to-face interviews two weeks after delivery. Face-to-face interviews were chosen since this method enhances participation, in particular by those with low education and migrants and since this method was also chosen in the WHO survey. A randomly selected subset of women was invited by their own midwife for study participation. The interview took 30 minute face-to-face interview with an independent interviewer. The interviews were conducted by ten trained independent interviewers and usually performed at the respondent’s home. Each interview covered all three phases of the maternal perinatal experience. Interviewees were invited to respond to all questions, yet never forced to. Of the different interview modes, face-to-face interviews were chosen as this mode reduces non-response, and possibly also non-response bias. The face-to-face mode was also the preferred one used for a large number of the MCS Study countries and in the World Health Survey.

### Data handling and analysis

Records were regarded ‘missing at the record level’ if all scores of all phases were missing. If women had responded partially, the responses were evaluated per phase.

If all the items of one phase were missing, this record was excluded from the analysis of that phase. This implies that occasionally respondents were excluded from one phase while they were included in the analysis of other phases. Missing items were excluded from analysis.

We investigated the responsiveness questions’ psychometric properties stratified for the antenatal phase (the period from the onset of pregnancy until the onset of delivery), the birth phase (actual delivery) and postpartum phase (covering the first ten days after childbirth). The data were analysed with Statistical Package of Social Sciences version 20.0 for Windows (IBM Corp. Released 2011. IBM SPSS Statistics for Windows, Version 20.0. Armonk, NY: IBM Corp).

### Sumscores

Unweighted sumscores per domain were calculated and transformed into 1–10 scale scores to enhance comparability among domains with different numbers of items. Transformation was done as score =1 + 9* ([sumscore – lowest sum possible)]/[largest sum possible – lowest sum possible]). E.g., a domain that contains 3 items each with a 5-point response mode, displays a possible score range from 3 to 15. The transformed sumscore would then be 1 + 9* ([sumscore - 3]/[15 – 3]). If sumscore in an individual would be 11, her transformed score would be 1 + 9*([3]/[3]) = 1 + 9*(8/12) = 7. This transformation procedure was repeated for each domain in each phase separately.

### Psychometric tests

The following psychometric properties of the ReproQ were evaluated: feasibility, construct validity, and discriminative validity. Feasibility was expressed as rates of missing items per domain. The literature provides little indication of acceptable survey response rates or inappropriate non-response rates. We selected missing item rates below 20% as acceptable as done for another study [21]. In addition, we compared missing rates per item for each phase to identify problematic single items. Furthermore, we compared missing rates per domain by age, education, race, communication and health utilization to check for biases by social groups.

Scores per domain, expressed as transformed 1–10 scale scores, and scores per item, given in 1–5 scale scores, were described in terms of mean, SD, range, floor and ceiling effects, and percentiles.

Reliability was assessed as internal consistency by using Cronbach’s alpha. Amidst varying standards in the literature, we considered 0.70 to be an acceptable alpha coefficient [26].

Average inter-item, average item-own scale and average item-other scale correlation were assessed with standardized correlation coefficients, with acceptable correlations defined as Pearson’s correlation coefficients (r) >0.40 [27]. We expected higher average inter-item and average inter-own scale correlations compared to average inter-other scale correlations.

Discriminative validity was assessed by comparing subgroups expected to differ in terms of responsiveness. It was hypothesized that women whose child was not admitted to the hospital would report better responsiveness outcomes than women whose child was hospitalized. The rationale behind this hypothesis is that women with less good clinical outcomes would be more critical on the non clinical aspects of care given. Differences in overall mean sum scores (adding all domains) were calculated and tested with Student t-tests per phase.

Construct validity was assessed as the domain structure of factor loadings obtained with exploratory factor analysis using the maximum likelihood method with oblique promax rotation of factor loadings, extracting eight (fixed) factors. This was done to explore whether the original domain structure relevant to the generic responsiveness concept was present after adapting the responsiveness concept to perinatal services. The average inter-item correlations were 0.49 for the antenatal phase, 0.58 for the birth phase and 0.63 for the post partum phase. Average inter-item correlations were relatively low for the domains ‘Prompt Attention ‘and ‘Quality of Basic Amenities’. The item-own scale correlations for each phase separately were considerably higher than most of the corresponding item-other scale correlations. The overall average item-own scale correlation was 0.56 for the antenatal phase, 0.68 for the birth phase and 0.73 for the post partum phase.

## Results

Of a total of 274 women who were identified for study participation 94 women could not be reached or they declined the invitation; many women could not be reached using the cell phone number they had provided; we were unable to differentiate with limited means whether they refused the call, changed phone number, or had provided the wrong number. Other reasons for non-participation included lack of time, and feeling at unease of having a stranger visit their home. 180 women (66%) agreed to be interviewed. Of these seven interviews (7/180, 4%) were cancelled and two interviews (2/180, 1%) were discontinued because the respondents did not speak Dutch with sufficient fluency and no translator could be made available. The remaining 171 interviews were used for analysis. The interviews took between 20 and 40 minutes. Table 2 describes the characteristics of the participants.

**Table 2:** **Characteristics of the participants**

**Variable**	**n**	**%**
Maternal age*		
<19 years	3	2%
20-25 years	15	9%
25-34 years	119	70%
>35 years	33	19%
Missing	1	1%
**Parity**		
Primiparous	97	57%
Multiparous	74	43%
**Ethnic background**		
Dutch	94	55%
Non dutch	74	43%
**Education**		
Low	6	4%
Middle	75	44%
High	90	53%
**Marital status**		
Single	30	18%
Relationship/married	141	82%
**Neighbourhood**		
Privileged neighbourhood	84	49%
Underprivileged neighbourhood	87	51%
**Proficiency (speaking) dutch**		
Good/excellent	152	89%
Weak/poor	18	11%
Missing	1	1%
**Care process**		
Start antenatal care with midwife. not referred	61	36%
Start antenatal care with midwife. referred during antenatal phase to gynaecologist	37	22%
Start antenatal care with midwife. referred during birth phase	57	33%
Start antenatal care with gynaecologist	16	9%
**Hospital admission of child**		
No admission	145	85%
Admission	26	15%

*Mean age 31 (95% CI 30.0-31.7).

The mean maternal age was 31 years (95% CI 30.3–31.7). The majority of mothers were primiparous (57%). A substantial proportion of mothers was of non-Dutch origin (43%) or lived in underprivileged neighborhoods (51%). Few had low education (4%) or were single (18%). Approximately 11% spoke weak/poor Dutch as judged by the interviewer. Referral to gynecologists had occurred in approximately 55% of women. Post partum hospital admission had occurred in 26 (15%) of all newborns.

Table 3 describes the missing rates per domain for each phase separately. The table also describes the maximum missing rate per item for that domain. The results for four women, with no response in the birth care phase, were excluded.

**Table 3:** **Missing item values and the maximum percentage missing per item, for each domain and perinatal phase**

	**Antenatal phase**		(N = 171)*		**Birth phase**		(N = 167)*		**Post partum phase**		(N = 171)*		**Total**		**(N = 509)**
**Domain**	**Total items**	**Missing items (n)**	**Missing per domain (%)**	**Missing per item; maximum (%)**	**Total items**	**Missing items (n)**	**Missing per domain (%)**	**Missing per item; maximum (%)**	**Total items**	**Missing items (n)**	**Missing per domain (%)**	**Missing per item; maximum (%)**	**Total items**	**Missing items (n)**	**Missing per domain (%)**
**Dignity**	513	7	1%	1.8%	835	21	3%	5.3%	855	55	6%	10.5%	2203	83	4%
**Autonomy**	513	57	11%	11.1%	501	57	11%	14.0%	855	112	13%	15.8%	1869	226	12%
**Confidentiality**	513	28	5%	7.0%	1002	88	9%	11.1%	1026	91	9%	11.7%	2541	207	8%
**Communication**	855	35	4%	6.4%	1002	49	5%	11.7%	1026	65	6%	14.6%	2883	149	5%
**Prompt Attention**	684	28	4%	8.8%	1169	111	9%	18.1%	684	58	8%	15.2%	2537	197	8%
**Social Consideration**	342	16	5%	4.1%	501	65	13%	22.8%	855	60	7%	12.3%	1698	141	8%
**Quality of basic amenities**	342	8	2%	1.8%	501	45	9%	11.1%	513	35	7%	5.3%	1356	88	6%
**Choice and Continuity**	513	45	9%	10.5%	1169	130	11%	31.6%	855	83	10%	14.6%	2537	258	10%
**Total**	4275	224	5%		6680	566	8%		6669	559	8%		17624	1349	8%

*For each phase seperately records of non-responders were excluded.

The average item missing rate over all phases was 8% (1,349 out of 17,624 questions). Missing rates per domain were all below the predefined threshold of 20%. Average missing rates across domains were highest in the birth phase (8%). Maximum item missing rates per domain ranged from 1.8% to 11.1% for the antenatal phase, from 5.3% to 31.6% for the birth phase, and from 5.3% to 14.6% for the post partum phase (see Additional file 1: Table S6 Appendix table for detailed description of all items). The highest item missing rate was for two questions relevant to the birth phase: ‘Able to be referred to a medical specialist during birth care’ (31.6%) and ‘Consideration of home situation when planning appointments/examinations during birth care’ (22.8%). Item missing pertained mainly to the birth care phase and rates were higher among women of Dutch origin. There were no differences in missing rates by age, educational level and health utilization.

Table 4 displays the transformed scores per domain and phase (1–10 scale). Mean transformed scores were negatively skewed (7.1–8.4) as were the median scores (7.2-7.8). Floor effects were observed for up to 0.6% of women responding to a set of items in a particular domain for a particular phase, while ceiling effects were observed for up to 24% of cases. Mean scores and ceiling effects differed most across the domains in the antenatal phase and least across the domains in the post partum phase.

**Table 4:** **Mean (SD) transformed score, range, percentage floor and ceiling response, and Cronbach's α for each domain and phase**

**Domain**											
	**No. of items**	Mean*	SD*	Range*		**% Floor**	**% Ceiling**	**25th % tile**	**50th % tile**	**75th % tile**	**Cronbach's α**
**Dignity**											
Antepartum phase	3	8.4	1.1	5.5	10.0	0.0%	21.6%	7.8	7.8	9.3	0.73
Birth phase	5	8.1	1.1	1.1	10.0	0.0%	11.7%	7.8	7.8	9.1	0.86
Post partum phase	5	7.9	1.3	3.3	10.0	0.0%	12.3%	7.8	7.8	8.2	0.87
**Autonomy**											
Antepartum phase	3	7.8	1.2	3.3	10.0	0.0%	8.2%	7.0	7.8	8.5	0.73
Birth phase	3	7.7	1.4	1.4	10.0	0.0%	8.8%	7.7	7.8	7.8	0.87
Post partum phase	5	7.5	1.7	1.9	10.0	0.0%	0.6%	7.3	7.8	7.8	0.94
**Confidentiality**											
Antepartum phase	3	8.0	1.1	4.0	10.0	0.0%	14.0%	7.8	7.8	8.5	0.82
Birth phase	6	7.8	1.4	1.4	10.0	0.0%	12.3%	7.8	7.8	7.8	0.78
Post partum phase	6	7.7	1.4	1.8	10.0	0.0%	13.5%	7.4	7.8	7.8	0.94
**Communication**											
Antepartum phase	5	7.7	1.2	3.3	10.0	0.0%	5.3%	7.3	7.8	8.2	0.80
Birth phase	6	7.8	1.3	1.3	10.0	0.0%	9.9%	7.4	7.8	8.1	0.92
Post partum phase	6	7.6	1.7	1.0	10.0	0.6%	11.7%	7.4	7.8	8.1	0.95
**Prompt attention**											
Antepartum phase	4	7.1	1.4	1.0	10.0	0.6%	2.3%	6.6	7.2	7.8	0.67
Birth phase	7	7.7	1.3	1.3	10.0	0.0%	7.0%	7.1	7.8	8.4	0.83
Post partum phase	4	7.7	1.7	1.0	10.0	0.6%	12.9%	7.2	7.8	8.9	0.89
**Social consideration**											
Antepartum phase	2	7.1	1.8	1.0	10.0	0.6%	8.2%	5.5	7.8	7.8	0.76
Birth phase	3	7.6	1.6	1.6	10.0	0.6%	11.1%	7.0	7.8	7.8	0.87
Post partum phase	5	7.8	1.4	3.3	10.0	0.0%	8.2%	7.3	7.8	8.7	0.84
**Quality of basic amenities**											
Antepartum phase	2	7.5	1.4	3.3	10.0	0.0%	10.5%	6.6	7.8	7.8	0.57
Birth phase	3	7.6	1.4	1.4	10.0	0.0%	8.2%	7.0	7.8	8.5	0.66
Post partum phase	3	7.4	1.5	1.8	10.0	0.0%	6.4%	7.0	7.8	7.8	0.62
**Choice and continuity**											
Antepartum phase	3	7.3	1.7	1.0	10.0	0.6%	7.0%	6.3	7.8	7.8	0.77
Birth phase	7	7.2	1.5	1.5	10.0	0.0%	5.3%	6.5	7.6	7.8	0.88
Post partum phase	5	7.1	1.7	1.0	10.0	0.6%	7.0%	6.4	7.8	7.8	0.89

*Transformed 1–10 scale scores were used.

The Cronbach’s alpha ranged from 0.57-0.82 for the antenatal phase, from 0.66-0.92 for the birth care phase and from 0.62-0.95 for the post partum phase. For all phases the domain ‘Quality of Basic Amenities’ had lowest alphas.

Mean overall sum scores were higher for women whose child was not admitted after childbirth: 61.8 (sd 7.4) versus 58.3 (sd 5.1) (p = 0.02) in the antenatal phase; 61.9 (sd 8.4) versus 57.9 (sd 7.7) (p = 0.06) in the birth phase; and 62.1 (sd 9.2) versus 55.2 (sd 13.0) (p = 0.01) in the post partum phase.

Eight factors corresponding to the domain structure of the WHO responsiveness concept explained 69% of the variance in the antenatal phase, 69% in the birth phase and 76% in the post partum phase. Table 5 shows the final results of the oblique promax rotated factor loadings of the birth phase (the patterns of the antenatal and post partum phase were similar). Items that were expected to belong to one domain are outlined. The rotated solution of grouped items generally confirmed the hypothesized domain taxonomy within the birth and post partum phase. For the antenatal phase however, the hypothesized domain taxonomy was less evident with regard to ‘Social Consideration’ and ‘Choice and Continuity’, which appeared to be associated with other domains.

**Table 5:** **Promax rotated factor solution for the birth phase**

	**Factor**								
**Factor name**	**Confidentiality**	**Choice**	**Dignity**	**Prompt attention**	**Autonomy**	**Communication**	**Quality of basic amenities**		**Unique variance**
Respect shown during examinations (midwife)	.032	.096	**.670**	-.004	-.053	-.004	-.178	.243	.305
Examination room suitable to provide privacy	.090	.018	**.792**	.018	-.163	-.221	.137	-.013	.277
Treated with respect (midwife)	-.103	-.138	**.871**	.037	-.059	.001	.057	.071	.310
Respect shown during examinations (nurse)	-.004	.031	**.768**	-.051	.092	.193	-.095	-.061	.267
Treated with respect (nurse)	-.056	-.103	**.568**	.032	.017	.244	.067	.029	.265
Involved in making a decision regarding your examinations or treatments	-.163	.041	.065	-.058	**.895**	-.051	.010	.080	.279
Able to refuse examinations or treatments	.011	-.029	-.162	.044	**1.009**	.059	-.102	-.006	.242
Asked permission before testing or starting treatment	.059	.187	-.033	-.046	**.693**	-.067	-.046	.003	.377
Protecting your confidentiality during consultations (midwife)	**.635**	.055	.135	-.003	.062	-.048	.100	.039	.234
Confidentiality kept on provided information (midwife)	**.773**	.076	.099	-.098	.055	.014	.078	-.064	.179
Confidentiality of patients’ medical records preserved (midwife)	**.843**	.063	.086	-.117	.065	-.020	-.070	.033	.161
Protecting your confidentiality during consultations (nurse)	**.548**	-.081	-.051	.091	-.192	-.126	.071	.156	.584
Confidentiality kept on provided information (nurse)	**.955**	-.079	-.066	.144	-.108	.113	-.023	-.050	.156
Confidentiality of patients’ medical records preserved (nurse)	**.872**	.012	-.160	.099	.046	.089	.003	.016	.140
Information clearly explained (midwife)	.236	-.221	-.072	.164	.225	.236	.000	.359	.281
Information about other treatment options (midwife)	.065	.057	-.022	-.155	.157	.181	.093	**.641**	.211
Encouraged to ask questions about diseases. treatment and care (midwife)	.105	.010	.131	.006	-.068	.201	-.059	**.694**	.233
Information clearly explained (nurse)	.032	-.139	-.041	.013	.057	**.720**	.085	.188	.234
Information about other treatment options (nurse)	.009	.079	-.062	-.078	.079	**.701**	.085	.225	.172
Encouraged to ask questions about diseases. treatment and care (nurse)	.065	.047	.124	-.036	-.090	**.699**	-.045	.227	.216
Experience of the waiting time when arriving on the place of delivery	.154	.031	-.102	**.743**	-.121	.012	-.051	.005	.423
Experience of the waiting time on examinations	-.108	.109	.030	**.683**	.003	-.001	-.096	.399	.257
Experience of the waiting time after you asked for help (midwife)	-.056	.196	.072	**.724**	-.068	-.101	-.031	-.014	.360
Accessibility by phone (midwife)	-.026	.013	.188	.082	.089	.099	.270	.080	.493
Travelling time to the place of birth	.082	-.144	.085	**.514**	.215	.038	.028	-.174	.463
Experience of the waiting time after you asked for help (nurse)	.012	.034	.030	**.406**	-.023	.118	.234	-.002	.387
Accessibility by phone (nurse)	.055	.164	-.020	.195	-.033	.237	.218	-.023	.402
Facilitate the support of relatives and friends (midwife)	.144	.025	.000	-.064	-.098	-.032	**.937**	-.017	.253
Consideration of home situation when planning appointments/examinations	.123	.101	.089	-.121	.190	.018	**.466**	.119	.253
Facilitate the support of relatives and friends (nurse)	-.091	.075	.050	.077	-.057	.219	**.690**	-.026	.287
Hygiene of the toilets and examination rooms.	-.068	-.086	.244	.167	.206	.108	.151	-.160	.503
Comfort of the examination rooms and waiting rooms	.157	-.002	.256	.303	.201	-.108	.005	-.241	.440
Quality of the food	-.078	.237	.143	.171	.193	-.153	-.039	-.028	.667
Able to choose own health care provider (midwife)	.119	**.611**	-.116	.124	.032	-.264	.144	.132	.383
Able to be referred to a medical specialist (midwife)	.267	.398	.127	-.165	.069	-.062	.014	.074	.404
Presence of different health care providers (midwife)	.062	**.622**	-.039	.056	.066	.128	-.096	.043	.295
Continuity of care by one health care provider (midwife)	.059	**.434**	.148	.179	.034	-.003	-.094	.119	.321
Able to choose own health care provider (nurse)	-.207	**.683**	-.123	.020	.077	-.062	.344	-.016	.327
Presence of different health care providers (nurse)	-.043	**.760**	-.002	-.029	-.076	**.495**	-.169	-.150	.269
Continuity of care of one health care provider (nurse)	-.045	**.492**	-.024	.049	-.101	**.558**	.065	-.131	.294

Total variance explained; 69%.

For each factor the rotated solution of grouped items are made bold.

## Discussion

With the support of both patients and health care providers, we adapted the WHO’s concept of responsiveness and the World Health Survey responsiveness module into the ReproQ instrument to measure responsiveness in the Dutch obstetric care system antenatally, during childbirth and post partum. ReproQ was administrated in a face-to-face interview context and appears to be a potential instrument for reporting perinatal service quality from the client's perspective. The perinatal responsiveness items grouped in the original eight domain based structure found in the World Health Survey and the World Health Survey questionnaire and appeared to be comprehensive, as judged by the experts. The ReproQ demonstrated satisfactory psychometric properties to describe the responsiveness outcomes of perinatal care in the Netherlands, with preliminary evidence on the questionnaire’s ability to discriminate between levels of non-clinical quality of care.

Particular strengths of adapting an existing WHO responsiveness concept and measurement approach are noted first. The eight domains were adopted a pre-existent conceptual structure that was identified in WHO’s review of the patient satisfaction and quality of care literature, which also included the examination of different survey instruments [15]. During this review, it was noted that the domains value is supported by human rights law which argues that the responsiveness features of a health system are important in their own right [14-16]. In contrast to patient satisfaction questionnaires, the concept of responsiveness aims to capture the patient’s actual experiences, since literature has shown that expectations strongly influence patient satisfaction. Expectations may be influenced by economic influences, political influences, prior experiences and socio-demographic characteristics [17-20]. Fourthly, the WHO concept of responsiveness represents an universal concept (e.g. suitable for developing and developed countries, different ethnicities, different care systems) which allows valid comparisons across different countries, ethnicities or health care systems [14,28]. The Responsiveness concept is challenged by a number of issues. Firstly, although responsiveness aims to measure the patient’s actual experience, it is still disturbed by at least some extent of ‘subjectivity’. Secondly, capturing responsiveness by a limited number of questions with fixed answering categories is quite challenging. Combining qualitative research and different (quantitative) survey techniques, one can produce a richer, more valid, and more reliable findings than when adopting qualitative or quantitative methods alone [29].

In spite of the existing strengths of the Responsiveness concept and measurement approach, our study contributes to addressing some of the challenges of the Responsiveness concept and its measurement approach. This includes whether it can truly be adapted to specific areas of health systems, like perinatal care, and elicit participation from specific groups of user interacting with specific health service. In particular, we found that users of perinatal services were interested in participating in the survey on non-clinical aspects of their care experience. Participation rates were equal or higher than the participation rates found in other perinatal satisfaction studies [30-32]. Participation rates were equal to participation rates found in surveys measuring similar domains of quality of care, and better than obtained by WHO’s Multi-Country Survey (MCS) study administered in the Netherlands in 2001 (59%) [21,25,33]. Comparisons are made with the MCS Study that was conducted in the Netherlands in 2001 as the questionnaire contained multiple items for each responsiveness domain, whereas the subsequent World Health Survey only contained one question per domain [14,21].

An optimal data collection method includes one with an explicit trade-off balance between cost and errors including nonsampling error, coverage error, nonresponse error and measurement error [34]. To ensure data quality we chose face-to-face interviews with an independent interviewer for data collection. Compared to self-administered forms face-to-face interviews perform better in terms of non-sampling and non-response error but may perform worse when sensitive questions are asked and are more costly [35]. Internet or web surveys are less costly and more time efficient but also have limitations especially including coverage error [36,37]. Mixed-mode approaches, combining the best of both worlds (being less costly and having less error than in a unimode approach) are very promising and should be considered [38].

The average item missing rates across domains was 8%, which according to literature can be considered acceptable. Within the framework of the MCS study, slightly lower overall missing rate was reported (5.0%) [39]. However, our survey dealt with a group of women who were extremely occupied with the challenging demands of new life, being interviewed post partum. Our survey focused on three phases of a specific health event, which may have been more cognitively demanding than the MCS study, which focused on reporting on an average experience in the previous 12 months, and was shorter (on average 25 minutes) [32,40,41]. As found in the MCS, the domain missing rate was highest for the domains of ‘Autonomy’ and ‘Choice and Continuity’ which are typically cognitively demanding domains. Across phases of perinatal care, the missing rate was highest for birth phase. But in general we found the proportion of missing rates per item to be similar across items. There were two items in the birth phase that had notably higher missing rates. Most likely this is the consequence of these items pointing to service events that do not always taking place. For this paper we excluded them from the analysis. We do not feel this hindered our ability to test the ReproQ psychometric properties, but have noted difficulties with these items for future surveys. Alternatively, when not all women experience all the events that can occur, different responsiveness scores may be presented for certain service events that occurred as well as in absence of those events.

The transformed scale scores were satisfactory. A floor effect was almost absent as is frequently the case in negatively skewed assessments of self-reported health or self-rated experiences of (maternity) care [30-32,42]. There was surprisingly less skewing towards use of the most positive category (ceiling effects) compared to other surveys e.g. in the MCS [32,40,41]. Comparisons of the domain scores across the three phases showed a non-uniform pattern, suggesting that respondents judged each phase separately as was intended by the questionnaire design.

Within each phase and for all domains, the questionnaire’s internal consistency was good. Cronbach’s Alpha coefficients in ReproQ were similar compared to the CAHPS and WHO surveys [21,25], except for the domain ‘Quality of Basic Amenities’ which showed poor alphas in all phases. This domain contained questions about sanitary hygiene, comfort of waiting room and quality of food. It can be argued that these elements of basic amenities were too diverse to achieve internal consistency (see Table 5) and one might improve reporting of results from the questionnaire by analyzing these items separately. The Pregnancy and Childbirth questionnaire (PSQ) covering personal treatment patient satisfaction outcomes for the antenatal and birth phase [43] showed higher Cronbach’s Alpha coefficients for the antenatal phase (0.89 vs.0.73) and for the birth phase (0.86 vs. 0.84). However, no predefined domain structure was used.

Overall, the taxonomy of domains from the WHO concept and measurement approach held for the adapted items in the ReproQ across all phases, although this taxonomy was weaker in the antenatal phase. This could possibly be due to factors such as; recall bias introduced by assessing all phases together, contamination by pregnancy outcome, focusing on one particular event or the heterogeneity in measurements since antenatal care consists of multiple visits. Underlying patterns are still to be explored. One may consider presenting a questionnaire on the antenatal phase within the antenatal phase, separately from a questionnaire on the birth and post partum phase. The total explained variance for the birth phase was higher in our study compared to the PCQ [43] (69% vs. 56%) as for the antenatal phase (69% vs. 53%).

The ability of the instrument to discriminate between good and less good experiences will be of paramount importance for its future use. We found some promising test results. The respondents clearly expressed different opinions on their experiences in the different phases of perinatal care. The non-uniform pattern of domain scores across the three phases suggested that respondents judged each phase separately as was intended by the questionnaire design. Furthermore discrimination between women whose infants were admitted to hospital subsequent to birth, was reflected in the lower sum scores across all phases. However, to test the difference in mean responsiveness of the birth phase between the mothers whose infant was hospitalised and the mothers whose infants were not hospitalised (mean difference: 3.8, pooled SD 6.5), at least 194 mothers had to be included in the analysis (type I error = 0.05 (two-sided), power = 0.80, control/case-ratio: 6/1). This implies that different responses on antenatal sum scores may reflect a true outcome on non-clinical aspects of care or may be contaminated by pregnancy outcomes. This again stresses the need to present a questionnaire on the antenatal phase separately.

Test-retest reliability was not performed in this stage. Reasons were to avoid the already large burden for the participants and to avoid associated potential recall bias effects due to having at this time a too demanding interview.

## Conclusions

Overall, our study found that ReproQ demonstrated satisfactory psychometric properties to describe the responsiveness outcomes of perinatal care in the Netherlands, with preliminary evidence supporting the questionnaire’s ability to discriminate between levels of non-clinical quality of care.’ In general, psychometric properties were in line with results obtained for other survey instruments that have been tested and promoted as part of quality assessment effort. In conclusion, given the lack of comparable instruments and the overall favorable study results, we feel that this unique adaptation of the WHO responsiveness questionnaire to evaluate the various phases of perinatal care has been relatively successful. With some minor adaptations as suggested throughout the discussion we believe that this questionnaire can be used to evaluate the quality of perinatal care in the Netherlands and elsewhere.

## Additional file

Additional file 1: Table S6. Appendix Missing values, mean(SD), range, percentage floor and ceiling given for each item separately.

## Abbreviations

WHO: World health organization
MCS: Multi-country survey
WHS: World health survey
PSQ: The pregnancy and childbirth questionnaire
